# Supplementary material for: Effect of Short-Term Restraint Stress on the Hypothalamic Transcriptome Profiles of Rats with Inherited Stress-Induced Arterial Hypertension (ISIAH) and Normotensive Wistar Albino Glaxo (WAG) Rats
Source: Int J Mol Sci. 2024 Jun 18;25(12):6680. doi: 10.3390/ijms25126680 (PMC11203755; doi:10.3390/ijms25126680)
Supplement: Supplementary file 1 [file ijms-25-06680-s001.zip › ijms-3015263-supplementary.pdf]

Supplementary Tables

Table of contents: page

Table S1: List and expression of ISIAH specific 113 DEGs: 1
Table S2: List and expression of WAG specific 85 DEGs: 5
Table S3: List and expression of 144 common ISIAH DEGs: 8
Table S4: List and expression of 144 common WAG DEGs: 12
Figure S1. Functional annotation of 10 ISIAH strain specific DEGs associated with Potassium ion transmembrane transport: 16
Figure S2. DEGs that altered transcription levels by 1.5-fold in the hypothalamus of only one of the rat strains: 17
Figure S3. Comparison of key DEG expression changes in the hypothalamus of ISIAH and WAG rats when exposed to restraint stress: 18

Supplementary Table S1. List and expression of ISIAH specific 113 DEGs

ISIAH_specific DEGs	GeneID	log2 Fold Change	padj	description
Adamts18	361412	-1,084	5,36E-03	ADAM metallopeptidase with thrombospondin type 1 motif, 18
Alg10	245960	-0,656	9,17E-04	ALG10, alpha-1,2-glucosyltransferase
Alox15	81639	1,236	1,98E-02	arachidonate 15-lipoxygenase
Angptl2	171100	-0,663	6,44E-05	angiopoietin-like 2
Aox3	493909	-0,719	5,75E-04	aldehyde oxidase 3
Apoa1	25081	0,754	2,24E-03	apolipoprotein A1
Ar	24208	-0,840	6,27E-03	androgen receptor
Arid5b	309728	-0,597	1,45E-04	AT-rich interaction domain 5B
Clcn5	25749	-0,761	5,75E-03	chloride voltage-gated channel 5
Cldn2	300920	-2,024	3,85E-02	claudin 2
Clec4a	474143	-0,835	1,01E-02	C-type lectin domain family 4, member A
Cmtm4	498902	-0,639	4,86E-04	CKLF-like MARVEL transmembrane domain containing 4
Col4a3	363265	-0,691	4,48E-03	collagen type IV alpha 3 chain
Col8a1	304021	-0,691	1,19E-02	collagen type VIII alpha 1 chain
Cxcl13	498335	-0,635	2,69E-02	C-X-C motif chemokine ligand 13
Cybb	66021	-0,778	1,27E-02	cytochrome b-245 beta chain
Cyp1b1	25426	-0,784	3,50E-03	cytochrome P450, family 1, subfamily b, polypeptide 1
Disc1	307940	-0,684	3,98E-02	DISC1 scaffold protein
Dok6	498898	-0,850	3,53E-03	docking protein 6
Egr4	25129	0,983	1,55E-03	early growth response 4
Epb41l4b	500464	-0,590	3,44E-04	erythrocyte membrane protein band 4.1 like 4B
F5	304929	-1,790	4,62E-02	coagulation factor V
Fblim1	362650	-0,677	1,51E-02	filamin binding LIM protein 1
Fndc11	499953	-0,697	4,93E-02	fibronectin type III domain containing 11

Fos	314322	0,760	2,94E-02	Fos proto-oncogene, AP-1 transcription factor subunit
Foxn3	314374	-0,619	1,64E-04	forkhead box N3
Frem2	310418	-0,894	3,31E-03	FRAS1 related extracellular matrix 2
Fscn2	303741	-0,745	3,28E-02	fascin actin-bundling protein 2, retinal
Gabrb2	25451	-0,621	1,19E-02	gamma-aminobutyric acid type A receptor subunit beta 2
Gfod1	306842	-0,626	2,50E-03	glucose-fructose oxidoreductase domain containing 1
Glra3	114516	-0,774	4,70E-02	glycine receptor, alpha 3
Gpr151	307475	2,207	2,51E-02	G protein-coupled receptor 151
Grm5	24418	-0,606	2,10E-04	glutamate metabotropic receptor 5
Gucy2d	79222	-1,028	1,51E-02	guanylate cyclase 2D, retinal
Hivep3	313557	-0,662	1,39E-05	HIVEP zinc finger 3
Hs6st3	364476	-0,672	4,42E-03	heparan sulfate 6-O-sulfotransferase 3
Itga1	25118	-0,782	2,71E-02	integrin subunit alpha 1
Itgal	308995	-0,587	1,20E-02	integrin subunit alpha L
Jmjd7	1E+08	-0,600	1,52E-02	jumonji domain containing 7
Kcna2	25468	-0,703	1,63E-02	potassium voltage-gated channel subfamily A member 2
Kcna3	29731	-0,663	4,83E-02	potassium voltage-gated channel subfamily A member 3
Kcne2	171138	-0,786	3,34E-03	potassium voltage-gated channel subfamily E regulatory subunit 2
Kcnj10	29718	-0,664	4,01E-02	potassium inwardly-rectifying channel, subfamily J, member 10
Kcnj13	94341	-0,910	3,69E-03	potassium inwardly-rectifying channel, subfamily J, member 13
Kcnmb1	29747	-0,874	3,08E-02	potassium calcium-activated channel subfamily M regulatory beta subunit 1
Kcnq2	170848	-0,710	2,23E-03	potassium voltage-gated channel subfamily Q member 2
Kcnv2	294065	-1,220	1,33E-02	potassium voltage-gated channel modifier subfamily V member 2
Kel	297025	0,883	2,82E-02	Kell metallo-endopeptidase (Kell blood group)
Kirrel1	310695	-0,722	2,56E-03	kirre like nephrin family adhesion molecule 1
Klf12	306110	-0,616	3,98E-02	Kruppel-like factor 12
Klf13	499171	-0,949	1,65E-03	Kruppel-like factor 13
Klrk1	24934	-0,996	4,67E-03	killer cell lectin like receptor K1
Lancl3	302540	-0,793	3,37E-02	LanC like 3
Maml3	310405	-0,601	1,91E-02	mastermind-like transcriptional coactivator 3
Map2	25595	-0,710	1,13E-02	microtubule-associated protein 2
Mas1	25153	-0,631	1,09E-02	MAS1 proto-oncogene, G protein-coupled receptor
Mbtps2	302705	-0,603	3,95E-03	membrane-bound transcription factor peptidase, site 2
Mob3b	366352	-0,680	4,98E-04	MOB kinase activator 3B
Nemp1	679504	-0,736	2,66E-02	nuclear envelope integral membrane protein 1
Nfe2l3	312331	1,122	2,50E-05	nuclear factor, erythroid 2-like 3

Nmnat2	289095	-0,590	5,33E-07	nicotinamide nucleotide adenylyltransferase 2
Nox4	85431	-0,737	2,85E-02	NADPH oxidase 4
Npr3	25339	-0,975	1,25E-02	natriuretic peptide receptor 3
Optc	304802	-0,843	3,77E-03	opticin
Palm2	1,04E+08	-0,680	1,72E-02	paralemmin 2
Pcdha2	116741	-0,781	7,09E-03	#Н/Д
Pcdhb21	307487	-0,885	4,68E-02	protocadherin beta 21
Pcdhga1	553129	-0,592	3,34E-02	protocadherin gamma subfamily A, 1
Pcdhga9	252895	-0,677	1,17E-02	protocadherin gamma subfamily A, 9
Pde11a	140928	-0,636	1,11E-02	phosphodiesterase 11A
Pde5a	171115	-0,605	4,75E-03	phosphodiesterase 5A
Pdpk1	81745	-0,655	2,61E-03	3-phosphoinositide dependent protein kinase-1
Phf11	361051	-0,606	3,83E-02	PHD finger protein 11
Pkp1	304822	-0,642	4,64E-05	plakophilin 1
Plcxd3	310358	-0,684	2,89E-05	phosphatidylinositol-specific phospholipase C, X domain containing 3
Pld5	289270	-0,773	6,10E-05	phospholipase D family, member 5
Pnpla1	361812	-0,601	4,18E-02	patatin-like phospholipase domain containing 1
Prkaa2	78975	-0,762	1,11E-02	protein kinase AMP-activated catalytic subunit alpha 2
Prlh	63850	1,030	4,65E-02	prolactin releasing hormone
Prox1	305066	-0,649	3,37E-04	prospero homeobox 1
Psca	680210	-1,043	3,54E-02	prostate stem cell antigen
Ptbp3	83515	-0,616	3,54E-03	polypyrimidine tract binding protein 3
Ptpn14	305064	-0,710	1,71E-02	protein tyrosine phosphatase, non-receptor type 14
Rasgrp4	170668	-0,611	2,85E-02	RAS guanyl releasing protein 4
RGD1305347	362576	-0,628	3,77E-02	similar to RIKEN cDNA 2610528J11
Rnf125	361296	-0,940	3,22E-02	ring finger protein 125
Scn4a	25722	-0,803	3,44E-07	sodium voltage-gated channel alpha subunit 4
Scn8a	29710	-0,644	1,58E-07	sodium voltage-gated channel alpha subunit 8
Siah3	692004	-0,948	1,84E-02	siah E3 ubiquitin protein ligase family member 3
Slc10a1	24777	-1,011	4,34E-02	solute carrier family 10 member 1
Slc16a14	316578	-0,713	1,65E-02	solute carrier family 16, member 14
Slc4a1	24779	1,231	2,55E-02	solute carrier family 4 member 1 (Diego blood group)
Slc4a8	315311	-0,656	4,01E-05	solute carrier family 4 member 8
Slc6a12	50676	-0,730	1,91E-04	solute carrier family 6 member 12
Slfn4	114247	-0,797	2,80E-04	schlafen family member 4
Sox6	293165	-0,713	1,01E-03	SRY-box transcription factor 6
Spata18	289586	-0,700	1,06E-02	spermatogenesis associated 18
Spata32	287747	-0,624	4,46E-02	spermatogenesis associated 32
Spry3	498159	-0,656	1,11E-02	sprouty RTK signaling antagonist 3
Srgap3	500287	-0,766	4,05E-03	SLIT-ROBO Rho GTPase activating protein 3
St8sia6	291325	-1,116	3,44E-02	ST8 alpha-N-acetyl-neuraminide alpha-2,8-sialyltransferase 6
Stk32a	364858	-0,861	8,44E-03	serine/threonine kinase 32A

Ston2	314349	-0,747	2,22E-02	stonin 2
Stox2	306459	-0,634	6,10E-05	storkhead box 2
Tekt4	302991	0,624	3,02E-09	tektin 4
Tgfbr3	29610	-0,617	4,49E-06	transforming growth factor beta receptor 3
Tnfsf10	246775	-0,952	3,51E-05	TNF superfamily member 10
Tnr	25567	-0,616	5,54E-05	tenascin R
Trim67	307938	-0,878	2,53E-02	tripartite motif-containing 67
Trim72	365377	-0,692	4,99E-04	tripartite motif containing 72
Trpc5	140933	-0,685	6,28E-04	transient receptor potential cation channel, subfamily C, member 5
Vof16	259227	-0,754	1,06E-02	ischemia related factor vof-16
Wnk3	317420	-0,588	7,27E-03	WNK lysine deficient protein kinase 3

Supplementary Table S2: List and expression of WAG specific 85 DEGs

WAG_specific DEGs	GeneID	log2 Fold Change	padj	description
Adgrg5	307645	1,187	3,83E-02	adhesion G protein-coupled receptor G5
Adra1b	24173	0,593	8,45E-03	adrenoceptor alpha 1B
Adra2b	24174	0,933	1,98E-02	adrenoceptor alpha 2B
Akap5	171026	-0,681	2,29E-02	A-kinase anchoring protein 5
Alkal2	679566	0,767	8,81E-04	ALK and LTK ligand 2
Aox2	316421	0,735	6,49E-03	aldehyde oxidase 2
Arrdc2	306344	0,709	8,01E-15	arrestin domain containing 2
Asb18	316614	1,153	2,20E-03	ankyrin repeat and SOCS box-containing 18
Atp2a1	116601	0,623	2,86E-03	ATPase sarcoplasmic/endoplasmic reticulum Ca2+ transporting 1
Baat	29725	-0,596	3,27E-02	bile acid CoA:amino acid N-acyltransferase
Best3	314847	1,031	2,24E-02	bestrophin 3
Car7	291819	1,378	4,25E-11	carbonic anhydrase 7
Cck	25298	0,818	8,82E-03	cholecystokinin
Ccl24	288593	-0,729	3,45E-02	C-C motif chemokine ligand 24
Ccl9	360579	-0,901	2,98E-02	chemokine (C-C motif) ligand 9
Ccn1	83476	-0,850	1,10E-03	cellular communication network factor 1
Cd101	310727	0,673	1,08E-03	CD101 molecule
Cga	116700	0,745	2,59E-07	glycoprotein hormones, alpha polypeptide
Chrna10	64574	-0,670	4,83E-02	cholinergic receptor nicotinic alpha 10 subunit
Chrna9	65024	1,003	2,76E-02	cholinergic receptor nicotinic alpha 9 subunit
Chst5	307859	0,705	1,02E-05	carbohydrate sulfotransferase 5
Cldn1	65129	0,642	7,98E-03	claudin 1
Cytl1	498392	0,614	1,63E-03	cytokine like 1
Eaf2	266787	1,029	3,42E-02	ELL associated factor 2
Epha1	312279	0,750	1,58E-02	Eph receptor A1
Errfi1	313729	0,621	1,98E-06	ERBB receptor feedback inhibitor 1
Esr1	24890	-0,763	4,84E-03	estrogen receptor 1
Fam163a	498257	0,644	3,50E-02	family with sequence similarity 163, member A
Fgf3	170633	-1,081	1,27E-02	fibroblast growth factor 3
Fzd5	317674	-0,627	8,32E-04	frizzled class receptor 5
Gabrd	29689	1,138	1,68E-02	gamma-aminobutyric acid type A receptor subunit delta
Gabrq	65187	-0,588	9,26E-07	gamma-aminobutyric acid type A receptor subunit theta
Gh1	24391	-7,283	8,09E-03	growth hormone 1
Hif3a	64345	1,398	4,62E-08	hypoxia inducible factor 3 subunit alpha
Il12rb2	171334	1,029	2,60E-03	interleukin 12 receptor subunit beta 2
Il20rb	501043	0,979	1,11E-02	interleukin 20 receptor subunit beta
Itgb3	29302	-0,989	4,10E-03	integrin subunit beta 3
Kcnj2	29712	-0,659	4,77E-03	potassium inwardly-rectifying channel, subfamily J, member 2

Kcnk15	156873	-0,637	1,84E-02	potassium two pore domain channel subfamily K member 15
Klra2	494194	-0,598	3,22E-02	killer cell lectin-like receptor, subfamily A, member 2
Lca5l	498065	-0,617	1,72E-03	lebercilin LCA5 like
Lilrb4	292594	-1,074	2,13E-02	leukocyte immunoglobulin like receptor B4
LOC310926	310926	1,451	1,26E-02	hypothetical protein LOC310926
Met	24553	-0,628	1,56E-02	MET proto-oncogene, receptor tyrosine kinase
Mlnr	252859	0,765	3,16E-03	motilin receptor
Mob3c	313511	-0,646	1,52E-02	MOB kinase activator 3C
Mybpc1	362867	1,312	2,94E-02	myosin binding protein C1
Nmu	63887	0,999	2,40E-09	neuromedin U
Nos2	24599	-0,963	4,90E-02	nitric oxide synthase 2
Nxf3	302591	-0,856	3,79E-02	nuclear RNA export factor 3
Pax4	83630	-0,681	3,26E-02	paired box 4
Pcdh12	116808	-0,791	1,63E-02	protocadherin 12
Pdk4	89813	1,169	2,90E-12	pyruvate dehydrogenase kinase 4
Phex	25512	-0,835	2,31E-02	phosphate regulating endopeptidase homolog, X-linked
Pou3f4	29589	-0,702	5,35E-05	POU class 3 homeobox 4
Ppm1j	295341	0,657	4,73E-03	protein phosphatase, Mg2+/Mn2+ dependent, 1J
Prkcd	170538	1,185	2,16E-02	protein kinase C, delta
Ramp3	56820	0,900	6,22E-03	receptor activity modifying protein 3
RGD1560775	501031	-0,679	2,79E-02	similar to RIKEN cDNA 4930579C12 gene
RT1-M1-4	294213	-1,007	3,45E-02	RT1 class I, locus M1, gene 4
Rxfp1	295144	-0,633	4,29E-02	relaxin family peptide receptor 1
Saxo2	293061	-0,612	2,87E-02	stabilizer of axonemal microtubules 2
Sema3a	29751	-0,756	7,95E-03	semaphorin 3A
Serpine1	24617	0,585	2,13E-04	serpin family E member 1
Sgk1	29517	1,197	7,82E-05	serum/glucocorticoid regulated kinase 1
Shisa8	315163	-0,678	4,84E-03	shisa family member 8
Shox2	25546	1,662	1,24E-04	short stature homeobox 2
Slc10a6	289459	0,731	3,81E-03	solute carrier family 10 member 6
Slc19a3	316559	1,232	2,66E-23	solute carrier family 19 member 3
Slitrk6	290467	0,633	1,07E-05	SLIT and NTRK-like family, member 6
Smpx	84416	1,417	3,47E-02	small muscle protein, X-linked
Smyd1	297333	-0,799	2,88E-02	SET and MYND domain containing 1
Synpo2	499702	0,720	4,41E-02	synaptopodin 2
Tcf7l2	679869	0,693	1,68E-02	transcription factor 7 like 2
Tent5b	313019	1,009	9,21E-03	terminal nucleotidyltransferase 5B
Tlr8	684440	-0,758	1,92E-02	toll-like receptor 8
Tmem140	362334	0,604	9,87E-09	transmembrane protein 140
Tmem252	361744	1,345	2,06E-19	transmembrane protein 252
Tnnc2	296369	0,608	2,92E-02	troponin C2, fast skeletal type
Treml1	501096	-0,945	3,54E-02	triggering receptor expressed on myeloid cells-like 1
Trib1	78969	0,611	3,38E-03	tribbles pseudokinase 1

Trpm5	365391	-1,216	4,51E-02	transient receptor potential cation channel, subfamily M, member 5
Tshb	25653	1,102	9,96E-08	thyroid stimulating hormone subunit beta
Ttk	315852	-0,799	5,99E-03	Ttk protein kinase
Wnt9b	303586	1,048	2,40E-02	Wnt family member 9B

Supplementary Table S3: List and expression of 144 common ISIAH DEGs

Common ISIAH_DEGs	GeneID	log2 Fold Change	padj	description
Abca4	310836	-0,690	7,51E-03	ATP binding cassette subfamily A member 4
Abcc1	24565	-0,605	2,97E-06	ATP binding cassette subfamily C member 1
Abcc6	81642	-0,612	3,13E-06	ATP binding cassette subfamily C member 6
Acr	24163	-0,755	4,92E-03	acrosin
Adamts12	294809	-0,723	4,70E-07	ADAM metallopeptidase with thrombospondin type 1 motif, 12
Alox12	287454	-0,716	3,07E-02	arachidonate 12-lipoxygenase, 12S type
Apln	58812	0,469	7,24E-06	apelin
Aspa	79251	0,368	3,76E-03	aspartoacylase
Atp2b4	29600	-1,000	2,75E-03	ATPase plasma membrane Ca2+ transporting 4
B3gntl1	367384	-0,667	1,11E-03	UDP-GlcNAc:betaGal beta-1,3-N-acetylglucosaminyltransferase-like 1
Bin2	366988	-0,435	4,58E-07	bridging integrator 2
Cables1	307585	0,392	6,96E-05	Cdk5 and Abl enzyme substrate 1
Cacna2d1	25399	-0,661	1,83E-02	calcium voltage-gated channel auxiliary subunit alpha2delta 1
Cacna2d2	300992	-0,621	2,52E-03	calcium voltage-gated channel auxiliary subunit alpha2delta 2
Ccdc117	498404	0,665	3,61E-03	coiled-coil domain containing 117
Cd180	294706	-1,194	1,70E-16	CD180 molecule
Cd274	499342	-0,665	1,51E-02	CD274 molecule
Cdkn1a	114851	0,934	2,04E-15	cyclin-dependent kinase inhibitor 1A
Cds2	114101	-0,609	9,12E-05	CDP-diacylglycerol synthase 2
Chordc1	315447	0,593	5,41E-08	cysteine and histidine rich domain containing 1
Chrm5	53949	-0,733	4,89E-03	cholinergic receptor, muscarinic 5
Chrna7	25302	-0,946	1,70E-06	cholinergic receptor nicotinic alpha 7 subunit
Clec10a	64195	0,417	6,72E-03	C-type lectin domain containing 10A
Cpne3	313087	-0,639	1,67E-03	copine 3
Creb5	500131	-0,635	2,61E-02	cAMP responsive element binding protein 5
Cryab	25420	0,670	2,22E-19	crystallin, alpha B
Cx3cr1	171056	-0,704	9,57E-11	C-X3-C motif chemokine receptor 1
Cxadr	89843	-0,644	1,38E-05	CXADR, Ig-like cell adhesion molecule
Cyp26b1	312495	-0,759	1,79E-04	cytochrome P450, family 26, subfamily b, polypeptide 1
Dab2	79128	-0,633	7,21E-05	DAB adaptor protein 2
Ddit4	140942	0,611	3,21E-03	DNA-damage-inducible transcript 4
Dhrs9	170635	-1,598	1,19E-04	dehydrogenase/reductase 9
Dio2	65162	0,536	1,50E-04	iodothyronine deiodinase 2
Dnajb1	361384	0,882	9,85E-04	DnaJ heat shock protein family (Hsp40) member B1
Edem3	289085	-0,591	1,09E-03	ER degradation enhancer, mannosidase alpha-like 3
Efnb2	306636	-0,734	6,98E-05	ephrin B2

Eva1a	500221	0,397	9,90E-03	eva-1 homolog A, regulator of programmed cell death
Evi2b	1,01E+08	-0,621	3,80E-03	ecotropic viral integration site 2B
Fibin	499856	0,420	2,26E-05	fin bud initiation factor homolog
Fkbp5	361810	0,785	1,98E-11	FKBP prolyl isomerase 5
Flvcr2	314323	-0,697	7,79E-05	FLVCR heme transporter 2
Fmo2	246245	0,690	3,09E-03	flavin containing dimethylaniline monoxygenase 2
Fndc3b	294925	-0,597	2,44E-02	fibronectin type III domain containing 3B
Fosb	1E+08	1,747	3,36E-19	FosB proto-oncogene, AP-1 transcription factor subunit
Fosl1	25445	1,233	7,82E-04	FOS like 1, AP-1 transcription factor subunit
Fosl2	25446	0,679	2,28E-02	FOS like 2, AP-1 transcription factor subunit
Frem1	298185	-0,901	8,30E-03	Fras1 related extracellular matrix 1
Gabrb3	24922	-0,652	2,88E-05	gamma-aminobutyric acid type A receptor subunit beta 3
Gpatch4	295228	0,575	1,98E-04	G patch domain containing 4
Gpd1	60666	1,020	1,68E-05	glycerol-3-phosphate dehydrogenase 1
Gpr101	317608	-0,678	3,37E-02	G protein-coupled receptor 101
Gpr34	554353	-0,558	2,94E-06	G protein-coupled receptor 34
Gpr4	308408	0,467	7,33E-03	G protein-coupled receptor 4
Grik3	298521	-0,715	2,92E-03	glutamate ionotropic receptor kainate type subunit 3
Grk3	25372	-0,726	2,80E-05	G protein-coupled receptor kinase 3
Hpd	29531	0,686	8,16E-04	4-hydroxyphenylpyruvate dioxygenase
Hspa1b	24472	2,881	1,76E-03	heat shock protein family A (Hsp70) member 1B
Hspb1	24471	1,030	3,04E-02	heat shock protein family B (small) member 1
Htr1a	24473	-0,575	3,95E-03	5-hydroxytryptamine receptor 1A
Idua	360904	-0,611	5,02E-11	alpha-L-iduronidase
Il17rd	498576	-0,759	3,19E-03	interleukin 17 receptor D
Il21r	308977	-0,600	3,50E-02	interleukin 21 receptor
Irf8	292060	-0,387	2,17E-02	interferon regulatory factor 8
Kantr	1,06E+08	-0,661	2,90E-03	KDM5C adjacent transcript
Kcnk9	84429	-0,977	6,44E-03	potassium two pore domain channel subfamily K member 9
Kdr	25589	-0,607	2,31E-13	kinase insert domain receptor
Kif1b	117548	-0,596	3,36E-03	kinesin family member 1B
Lims2	361303	0,590	9,11E-05	LIM zinc finger domain containing 2
Lmod2	296935	1,066	1,69E-02	leiomodin 2
LOC100362783	1E+08	0,805	3,94E-04	Uncharacterized protein C7orf61 homolog
Lonrf3	298322	0,573	1,92E-05	LON peptidase N-terminal domain and ring finger 3
Lrg1	367455	0,477	1,20E-02	leucine-rich alpha-2-glycoprotein 1
Lrrc58	303919	-0,608	3,37E-03	leucine rich repeat containing 58
Lyve1	293186	0,565	6,26E-04	lymphatic vessel endothelial hyaluronan receptor 1
Maff	366960	0,590	3,12E-04	MAF bZIP transcription factor F
Map3k6	313022	1,211	6,69E-05	mitogen-activated protein kinase kinase kinase 6

Mapkbp1	362197	-0,656	4,45E-05	mitogen activated protein kinase binding protein 1
Mlxipl	171078	-0,464	3,77E-03	MLX interacting protein-like
Mpeg1	64552	-0,610	1,63E-06	macrophage expressed 1
Mt2A	689415	0,655	1,68E-02	metallothionein 2A
Nav3	314814	-0,599	5,64E-10	neuron navigator 3
Ncoa2	83724	-0,819	2,25E-04	nuclear receptor coactivator 2
Ndst4	362035	-0,833	6,03E-04	N-deacetylase and N-sulfotransferase 4
Nfib	29227	-0,666	1,46E-02	nuclear factor I/B
Nfix	81524	-0,601	4,10E-04	nuclear factor I X
Niban1	63912	-0,767	2,35E-02	niban apoptosis regulator 1
Nos1	24598	-0,816	1,59E-02	nitric oxide synthase 1
Npas4	266734	1,087	2,92E-02	neuronal PAS domain protein 4
Nr2c2	50659	-0,611	5,60E-03	nuclear receptor subfamily 2, group C, member 2
Nrp2	81527	-0,588	4,60E-04	neuropilin 2
Opcml	116597	-0,627	2,72E-04	opioid binding protein/cell adhesion molecule-like
P2ry13	310444	-0,613	1,18E-04	purinergic receptor P2Y13
P2ry4	63843	-1,139	4,35E-02	pyrimidinergic receptor P2Y4
Pak3	29433	-0,664	4,31E-05	p21 (RAC1) activated kinase 3
Pcdh11x	317204	-0,611	2,50E-02	protocadherin 11 X-linked
Pcdh7	360942	-0,590	4,28E-04	protocadherin 7
Pcdhga2	498846	-0,611	1,05E-04	protocadherin gamma subfamily A, 2
Pex11a	85249	0,463	8,20E-06	peroxisomal biogenesis factor 11 alpha
Pgap1	316400	-0,618	1,01E-03	post-GPI attachment to proteins inositol deacylase 1
Pgr15l	296867	-0,516	3,63E-02	G protein-coupled receptor 15-like
Pik3ap1	294048	-0,625	3,00E-03	phosphoinositide-3-kinase adaptor protein 1
Pla2g3	289733	0,781	1,41E-03	phospholipase A2, group III
Plag1	297804	-0,623	1,82E-02	PLAG1 zinc finger
Plcb2	85240	-0,431	3,41E-02	phospholipase C, beta 2
Plek	364206	-0,607	3,49E-07	pleckstrin
Plekhf1	308543	0,521	6,54E-04	pleckstrin homology and FYVE domain containing 1
Pou2f2	117058	-1,003	1,56E-05	POU class 2 homeobox 2
Ppargc1a	83516	-0,630	1,31E-04	PPARG coactivator 1 alpha
Ppp1r9a	84685	-0,666	7,98E-04	protein phosphatase 1, regulatory subunit 9A
Pspn	25525	0,743	1,99E-02	persephin
Ptch1	89830	-0,644	8,17E-08	patched 1
Rasgrf2	114513	-0,720	2,22E-07	RAS protein-specific guanine nucleotide-releasing factor 2
Rasgrp3	313874	-0,662	8,28E-11	RAS guanyl releasing protein 3
Rin3	314397	0,595	1,89E-10	Ras and Rab interactor 3
Scml4	309859	-0,360	1,78E-03	Scm polycomb group protein like 4
Scrt2	366229	0,654	3,04E-06	scratch family transcriptional repressor 2
Slc16a2	259248	-0,603	2,27E-03	solute carrier family 16 member 2
Slc4a4	84484	-0,674	4,77E-03	solute carrier family 4 member 4

Slc7a14	499587	-0,947	4,44E-04	solute carrier family 7, member 14
Slc7a2	64554	-0,775	1,46E-03	solute carrier family 7 member 2
Smad9	85435	-0,645	2,68E-02	SMAD family member 9
Sned1	316638	-0,992	8,46E-07	sushi, nidogen and EGF-like domains 1
Spata13	305938	-0,844	4,58E-04	spermatogenesis associated 13
Srxn1	296271	0,530	8,97E-16	sulfiredoxin 1
Sult1a1	83783	0,436	5,79E-04	sulfotransferase family 1A member 1
Sv2c	29643	-0,823	8,85E-04	synaptic vesicle glycoprotein 2c
Tanc2	303599	-0,695	6,65E-05	tetratricopeptide repeat, ankyrin repeat and coiled-coil containing 2
Tenm2	117242	-0,602	4,63E-07	teneurin transmembrane protein 2
Tenm4	308831	-0,733	7,10E-06	teneurin transmembrane protein 4
Tgfbr2	81810	-0,667	4,75E-04	transforming growth factor, beta receptor 2
Tinagl1	94174	0,549	3,51E-07	tubulointerstitial nephritis antigen-like 1
Tlr2	310553	-0,544	2,71E-05	toll-like receptor 2
Tmc7	499254	-0,868	1,14E-08	transmembrane channel-like 7
Tmem119	304581	0,528	2,88E-07	transmembrane protein 119
Tnfrsf11a	498206	0,821	2,99E-09	TNF receptor superfamily member 11A
Tnfrsf12a	302965	0,558	1,41E-06	TNF receptor superfamily member 12A
Tppp	361466	-0,676	6,21E-03	tubulin polymerization promoting protein
Tsc22d3	83514	0,550	4,67E-13	TSC22 domain family, member 3
Ttll10	298692	-0,679	1,16E-02	tubulin tyrosine ligase like 10
Tub	25609	-0,588	7,06E-04	TUB bipartite transcription factor
Unc5d	306534	-0,690	7,74E-05	unc-5 netrin receptor D
Xylt1	64133	-0,648	5,75E-04	xylosyltransferase 1
Zbtb16	353227	1,228	9,41E-07	zinc finger and BTB domain containing 16
Zim1	308322	-0,630	1,16E-02	zinc finger, imprinted 1

Supplementary Table S4: List and expression of 144 common WAG DEGs

Common WAG_DEGs	GeneID	log2 Fold Change	padj	description
Abca4	310836	-0,466	2,73E-02	ATP binding cassette subfamily A member 4
Abcc1	24565	-0,400	1,63E-07	ATP binding cassette subfamily C member 1
Abcc6	81642	-0,447	1,51E-07	ATP binding cassette subfamily C member 6
Acr	24163	-0,764	4,95E-03	acrosin
Adamts12	294809	-0,289	2,41E-02	ADAM metallopeptidase with thrombospondin type 1 motif, 12
Alox12	287454	-0,779	8,06E-03	arachidonate 12-lipoxygenase, 12S type
Apln	58812	0,715	6,87E-24	apelin
Aspa	79251	0,595	2,06E-19	aspartoacylase
Atp2b4	29600	-0,613	2,65E-08	ATPase plasma membrane Ca2+ transporting 4
B3gntl1	367384	-0,389	2,33E-02	UDP-GlcNAc:betaGal beta-1,3-N-acetylglucosaminyltransferase-like 1
Bin2	366988	-0,817	1,36E-20	bridging integrator 2
Cables1	307585	0,615	3,85E-32	Cdk5 and Abl enzyme substrate 1
Cacna2d1	25399	-0,411	1,69E-04	calcium voltage-gated channel auxiliary subunit alpha2delta 1
Cacna2d2	300992	-0,407	3,73E-07	calcium voltage-gated channel auxiliary subunit alpha2delta 2
Ccdc117	498404	0,348	1,74E-04	coiled-coil domain containing 117
Cd180	294706	-1,614	8,45E-32	CD180 molecule
Cd274	499342	-0,463	3,06E-02	CD274 molecule
Cdkn1a	114851	1,352	4,53E-64	cyclin-dependent kinase inhibitor 1A
Cds2	114101	-0,327	5,19E-03	CDP-diacylglycerol synthase 2
Chordc1	315447	0,460	8,80E-11	cysteine and histidine rich domain containing 1
Chrm5	53949	-0,463	6,71E-03	cholinergic receptor, muscarinic 5
Chrna7	25302	-0,675	8,21E-05	cholinergic receptor nicotinic alpha 7 subunit
Clec10a	64195	0,666	2,73E-05	C-type lectin domain containing 10A
Cpne3	313087	-0,364	2,19E-02	copine 3
Creb5	500131	-0,664	2,42E-04	cAMP responsive element binding protein 5
Cryab	25420	0,824	4,04E-63	crystallin, alpha B
Cx3cr1	171056	-0,441	8,04E-11	C-X3-C motif chemokine receptor 1
Cxadr	89843	-0,573	2,75E-05	CXADR, Ig-like cell adhesion molecule
Cyp26b1	312495	-0,801	2,79E-03	cytochrome P450, family 26, subfamily b, polypeptide 1
Dab2	79128	-0,271	3,22E-02	DAB adaptor protein 2
Ddit4	140942	0,646	1,57E-04	DNA-damage-inducible transcript 4
Dhrs9	170635	-1,398	1,92E-03	dehydrogenase/reductase 9
Dio2	65162	0,939	3,93E-19	iodothyronine deiodinase 2
Dnajb1	361384	0,453	4,23E-12	DnaJ heat shock protein family (Hsp40) member B1
Edem3	289085	-0,397	3,01E-04	ER degradation enhancer, mannosidase alpha-like 3
Efnb2	306636	-0,558	7,33E-06	ephrin B2

Eva1a	500221	0,768	1,38E-20	eva-1 homolog A, regulator of programmed cell death
Evi2b	1,01E+08	-0,682	3,32E-03	ecotropic viral integration site 2B
Fibin	499856	0,643	2,20E-15	fin bud initiation factor homolog
Fkbp5	361810	1,382	1,24E-88	FKBP prolyl isomerase 5
Flvcr2	314323	-0,587	3,77E-04	FLVCR heme transporter 2
Fmo2	246245	0,965	9,20E-09	flavin containing dimethylaniline monoxygenase 2
Fndc3b	294925	-0,398	6,91E-03	fibronectin type III domain containing 3B
Fosb	1E+08	1,226	1,50E-03	FosB proto-oncogene, AP-1 transcription factor subunit
Fosl1	25445	1,699	2,15E-06	FOS like 1, AP-1 transcription factor subunit
Fosl2	25446	0,744	3,49E-03	FOS like 2, AP-1 transcription factor subunit
Frem1	298185	-0,573	4,03E-02	Fras1 related extracellular matrix 1
Gabrb3	24922	-0,405	2,19E-03	gamma-aminobutyric acid type A receptor subunit beta 3
Gpatch4	295228	1,009	5,51E-40	G patch domain containing 4
Gpd1	60666	1,677	1,09E-134	glycerol-3-phosphate dehydrogenase 1
Gpr101	317608	-0,544	1,11E-03	G protein-coupled receptor 101
Gpr34	554353	-1,030	7,02E-38	G protein-coupled receptor 34
Gpr4	308408	0,733	1,77E-06	G protein-coupled receptor 4
Grik3	298521	-0,406	3,98E-04	glutamate ionotropic receptor kainate type subunit 3
Grk3	25372	-0,389	1,93E-04	G protein-coupled receptor kinase 3
Hpd	29531	0,616	1,40E-03	4-hydroxyphenylpyruvate dioxygenase
Hspa1b	24472	1,006	1,72E-08	heat shock protein family A (Hsp70) member 1B
Hspb1	24471	0,479	2,64E-05	heat shock protein family B (small) member 1
Htr1a	24473	-0,639	9,84E-06	5-hydroxytryptamine receptor 1A
Idua	360904	-0,377	2,94E-07	alpha-L-iduronidase
Il17rd	498576	-0,655	1,92E-02	interleukin 17 receptor D
Il21r	308977	-0,659	5,56E-03	interleukin 21 receptor
Irf8	292060	-0,606	4,24E-09	interferon regulatory factor 8
Kantr	1,06E+08	-0,496	7,83E-03	KDM5C adjacent transcript
Kcnk9	84429	-0,444	2,19E-03	potassium two pore domain channel subfamily K member 9
Kdr	25589	-0,565	4,42E-10	kinase insert domain receptor
Kif1b	117548	-0,307	2,04E-02	kinesin family member 1B
Lims2	361303	1,019	5,02E-27	LIM zinc finger domain containing 2
Lmod2	296935	1,003	5,19E-03	leiomodin 2
LOC100362783	1E+08	0,437	2,30E-02	Uncharacterized protein C7orf61 homolog
Lonrf3	298322	0,769	1,57E-15	LON peptidase N-terminal domain and ring finger 3
Lrg1	367455	0,691	8,56E-11	leucine-rich alpha-2-glycoprotein 1
Lrrc58	303919	-0,480	3,06E-04	leucine rich repeat containing 58
Lyve1	293186	0,878	2,87E-10	lymphatic vessel endothelial hyaluronan receptor 1
Maff	366960	0,815	4,57E-09	MAF bZIP transcription factor F
Map3k6	313022	1,183	2,15E-13	mitogen-activated protein kinase kinase kinase 6

Mapkbp1	362197	-0,353	9,49E-06	mitogen activated protein kinase binding protein 1
Mlxipl	171078	-0,607	1,07E-06	MLX interacting protein-like
Mpeg1	64552	-0,289	2,70E-08	macrophage expressed 1
Mt2A	689415	0,592	1,66E-03	metallothionein 2A
Nav3	314814	-0,338	3,22E-04	neuron navigator 3
Ncoa2	83724	-0,356	1,01E-02	nuclear receptor coactivator 2
Ndst4	362035	-0,441	4,46E-02	N-deacetylase and N-sulfotransferase 4
Nfib	29227	-0,400	4,13E-02	nuclear factor I/B
Nfix	81524	-0,254	3,35E-02	nuclear factor I X
Niban1	63912	-0,577	2,71E-02	niban apoptosis regulator 1
Nos1	24598	-0,499	3,80E-04	nitric oxide synthase 1
Npas4	266734	-0,674	2,47E-03	neuronal PAS domain protein 4
Nr2c2	50659	-0,333	2,04E-02	nuclear receptor subfamily 2, group C, member 2
Nrp2	81527	-0,396	5,40E-05	neuropilin 2
Opcml	116597	-0,363	1,06E-02	opioid binding protein/cell adhesion molecule-like
P2ry13	310444	-0,464	4,54E-03	purinergic receptor P2Y13
P2ry4	63843	-0,765	3,32E-02	pyrimidinergic receptor P2Y4
Pak3	29433	-0,392	4,72E-02	p21 (RAC1) activated kinase 3
Pcdh11x	317204	-0,688	7,82E-05	protocadherin 11 X-linked
Pcdh7	360942	-0,416	1,55E-05	protocadherin 7
Pcdhga2	498846	-0,432	4,74E-05	protocadherin gamma subfamily A, 2
Pex11a	85249	0,740	1,72E-39	peroxisomal biogenesis factor 11 alpha
Pgap1	316400	-0,314	3,70E-02	post-GPI attachment to proteins inositol deacylase 1
Pgr15l	296867	-0,786	9,34E-06	G protein-coupled receptor 15-like
Pik3ap1	294048	-0,952	7,63E-08	phosphoinositide-3-kinase adaptor protein 1
Pla2g3	289733	1,856	1,65E-45	phospholipase A2, group III
Plag1	297804	-0,495	1,52E-02	PLAG1 zinc finger
Plcb2	85240	-0,596	2,20E-04	phospholipase C, beta 2
Plek	364206	-0,896	1,03E-13	pleckstrin
Plekhf1	308543	0,898	8,82E-25	pleckstrin homology and FYVE domain containing 1
Pou2f2	117058	-0,556	2,19E-03	POU class 2 homeobox 2
Ppargc1a	83516	-0,496	4,50E-04	PPARG coactivator 1 alpha
Ppp1r9a	84685	-0,446	2,52E-03	protein phosphatase 1, regulatory subunit 9A
Pspn	25525	0,493	4,66E-02	persephin
Ptch1	89830	-0,821	1,21E-30	patched 1
Rasgrf2	114513	-0,277	3,57E-03	RAS protein-specific guanine nucleotide-releasing factor 2
Rasgrp3	313874	-0,620	1,32E-17	RAS guanyl releasing protein 3
Rin3	314397	1,068	5,76E-48	Ras and Rab interactor 3
Scml4	309859	-0,608	9,87E-09	Scm polycomb group protein like 4
Scrt2	366229	0,645	4,68E-06	scratch family transcriptional repressor 2
Slc16a2	259248	-0,289	2,09E-02	solute carrier family 16 member 2
Slc4a4	84484	-0,350	3,07E-04	solute carrier family 4 member 4

Slc7a14	499587	-0,453	7,10E-03	solute carrier family 7, member 14
Slc7a2	64554	-0,454	8,76E-03	solute carrier family 7 member 2
Smad9	85435	-0,486	3,86E-02	SMAD family member 9
Sned1	316638	-0,383	2,60E-02	sushi, nidogen and EGF-like domains 1
Spata13	305938	-0,418	4,77E-03	spermatogenesis associated 13
Srxn1	296271	0,725	1,30E-38	sulfiredoxin 1
Sult1a1	83783	0,629	1,82E-22	sulfotransferase family 1A member 1
Sv2c	29643	-0,522	9,70E-03	synaptic vesicle glycoprotein 2c
Tanc2	303599	-0,259	4,31E-02	tetratricopeptide repeat, ankyrin repeat and coiled-coil containing 2
Tenm2	117242	-0,280	1,76E-04	teneurin transmembrane protein 2
Tenm4	308831	-0,374	7,75E-04	teneurin transmembrane protein 4
Tgfbr2	81810	-0,268	2,82E-02	transforming growth factor, beta receptor 2
Tinagl1	94174	0,751	9,21E-21	tubulointerstitial nephritis antigen-like 1
Tlr2	310553	-0,805	1,53E-11	toll-like receptor 2
Tmc7	499254	-0,718	1,75E-06	transmembrane channel-like 7
Tmem119	304581	0,649	3,07E-28	transmembrane protein 119
Tnfrsf11a	498206	1,185	2,02E-70	TNF receptor superfamily member 11A
Tnfrsf12a	302965	0,590	2,12E-09	TNF receptor superfamily member 12A
Tppp	361466	-0,360	5,41E-04	tubulin polymerization promoting protein
Tsc22d3	83514	0,739	2,98E-37	TSC22 domain family, member 3
Ttll10	298692	-0,861	1,05E-03	tubulin tyrosine ligase like 10
Tub	25609	-0,304	3,25E-04	TUB bipartite transcription factor
Unc5d	306534	-0,456	2,39E-03	unc-5 netrin receptor D
Xylt1	64133	-0,259	1,99E-02	xylosyltransferase 1
Zbtb16	353227	2,046	2,79E-52	zinc finger and BTB domain containing 16
Zim1	308322	-0,487	2,71E-03	zinc finger, imprinted 1

Supplementary Figures

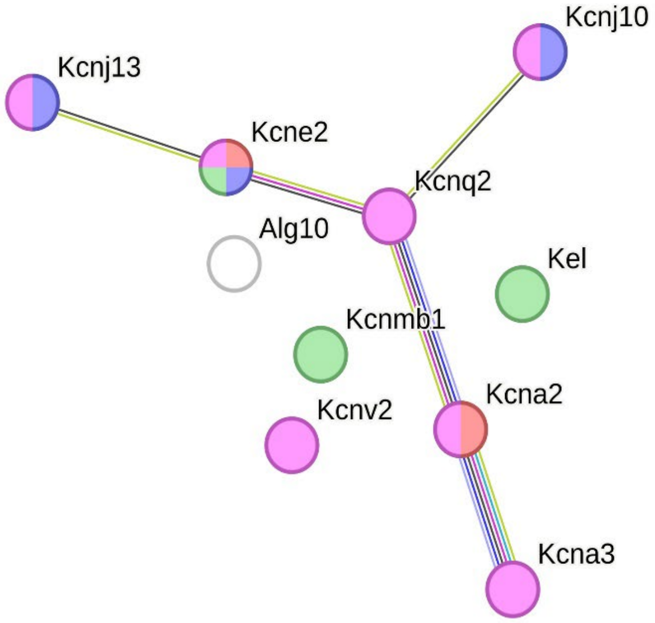

Biological Process (Gene Ontology)

GO-term	description	count in network	false discovery rate
GO:0097623	Potassium ion export across plasma membrane	2 of 17	0.0171
GO:1990573	Potassium ion import across plasma membrane	3 of 42	0.00072
GO:1901379	Regulation of potassium ion transmembrane transport	3 of 94	0.0070
GO:0005249	Voltage-gated potassium channel activity	7 of 96	6.44e-12

Figure S1. Functional annotation of 10 ISIAH strain specific DEGs associated with Potassium ion transmembrane transport. Edges represent protein–protein associations. Purple lines indicate experimentally determined interactions; blue lines denote known interactions from curated databases; dark blue lines represent gene co-occurrence; black lines indicate coexpression; green lines represent results of text mining. Protein-protein interaction (PPI) enrichment p-value: 0.000192.

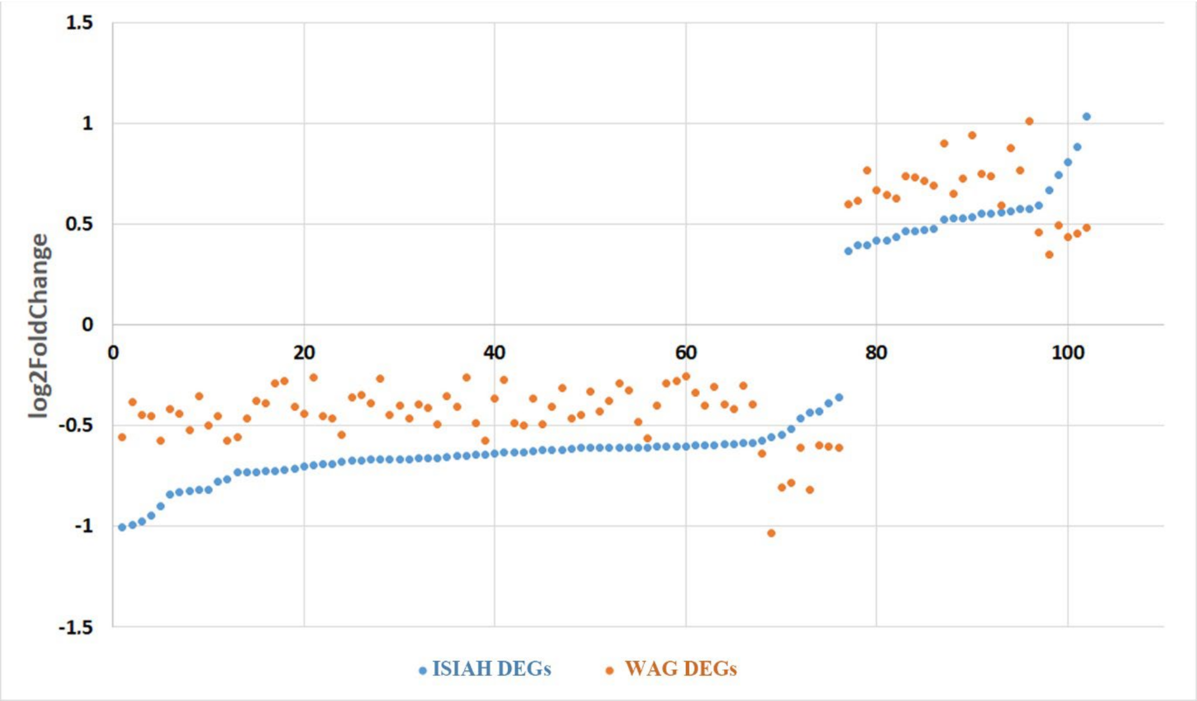

Figure S2. DEGs that altered transcription levels by 1.5-fold in the hypothalamus of only one of the rat strains.

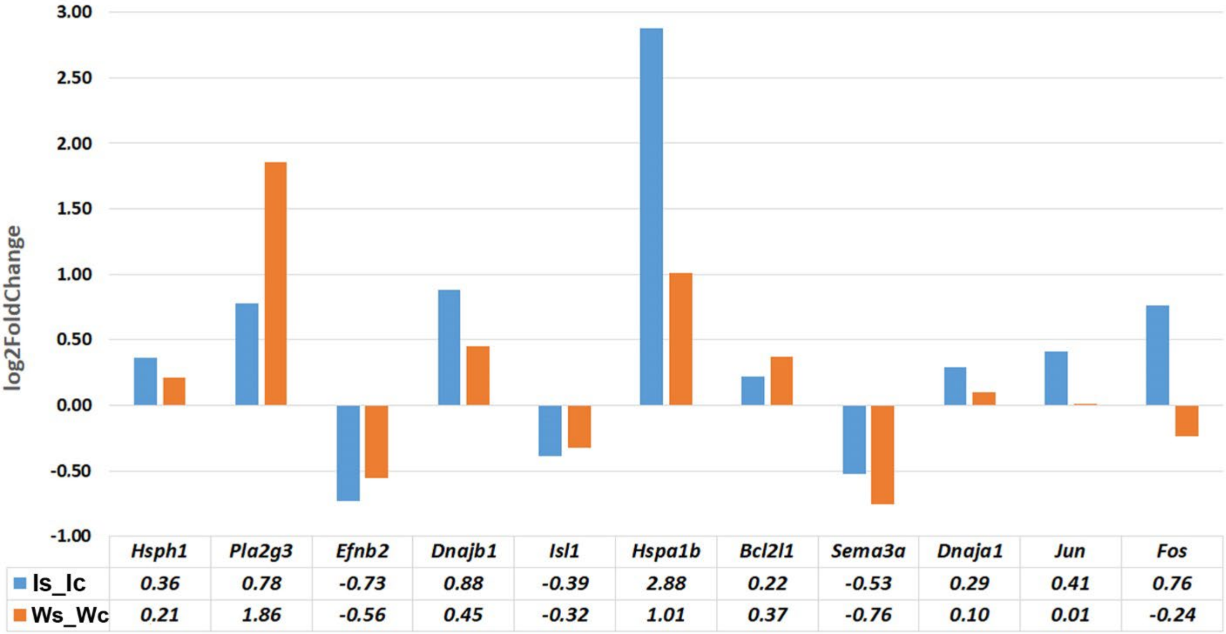

	Hsph1	*Pla2g3*	*Efnb2*	*Dnajb1*	*Isl1*	*Hspa1b*	*Bcl2l1*	*Sema3a*	*Dnaja1*	*Jun*	*Fos*
Is_Ic	*0.36*	*0.78*	*-0.73*	*0.88*	*-0.39*	*2.88*	*0.22*	*-0.53*	*0.29*	*0.41*	*0.76*
Ws_Wc	*0.21*	*1.86*	*-0.56*	*0.45*	*-0.32*	*1.01*	*0.37*	*-0.76*	*0.10*	*0.01*	*-0.24*

Figure S3. Comparison of key DEG expression changes in the hypothalamus of ISIAH and WAG rats when exposed to restraint stress. Ic, ISIAH_control; Wc, WAG_control; Is, ISIAH_stress; Ws, WAG_stress;
